# Sustainable conversion of polyethylene plastic bottles into terephthalic acid, synthesis of coated MIL-101 metal–organic framework and catalytic degradation of pollutant dyes

**DOI:** 10.1038/s41598-024-60363-5

**Published:** 2024-06-04

**Authors:** Fujiang Zhou, Danfeng He, Guojian Ren, Hossein Yarahmadi

**Affiliations:** 1 College of Science, Qiongtai Normal University, Haikou, 571100 Hainan China; 2 Key Laboratory of Advanced Materials of Tropical Island Resources, Ministry of Education, School of Chemistry and Chemical Engineering, Hainan University, Haikou, 570228 Hainan China; 3 Department of Chemical Engineering, Sirjan University of Technology, Sirjan, Iran

**Keywords:** MOF, PET, Degradation, Colored organic pollutant, Catalyst, Environmental chemistry, Nanoparticles

## Abstract

Persistent environmental colored compounds, resistant to biodegradation, accumulate and harm eco-systems. Developing effective methods to break down these pollutants is crucial. This study introduces Ag-MIL-101 (Ag-MIL-101) as a composite and reusable catalyst that efficiently degrades specific colored organic pollutants (COPs) like Methylene blue (MB), 4-Nitrophenol (4-NP), and 4-Nitroaniline (4-NA) using sodium borohydride at room temperature. The MIL-101 was synthesized using Terephthalic acid (TPA) derived from the degradation of Polyethylene Terephthalate (PET) plastic waste, with the assistance of zinc chloride. To further investigation, the kinetics of degradation reaction was studied under optimized conditions in the presence of Ag-MIL-101 as catalyst. Our results demonstrated the remarkable efficiency of the degradation process, with over 93% degradation achieved within just 8 min. The catalyst was characterized using FTIR, XRD, FESEM, and TEM. In this study, the average particle size of Ag-MIL-101 was determined using SEM and XRD analysis. These methods allow us to accurately and precisely determine the particle size. We determined the reaction rate constants for the degradation of each COP using a pseudo first-order kinetic equation, with values of 0.585, 0.597 and 0.302 min^−1^ for MB, 4-NP, and 4-NA, respectively. We also evaluated the recyclability of the catalyst and found that it could be reused for up to three cycles with only a slight decrease in efficiency (10–15%). Overall, our findings highlight the promising application of Ag-MIL-101 as an effective catalyst for the degradation of COPs, emphasizing the importance of optimizing reaction conditions to achieve enhanced efficiency.

## Introduction

Releasing 80% of untreated sewage globally poses health risks. Rapid industrial development has generated hazardous waste that remains undecomposed. Pollutants like heavy metals and toxic organic compounds (e.g., methylene blue, nitro phenols) can alter water chemistry^1,2^. This problem stems from various industries (e.g., dye, pigment, textile) releasing organic and soluble pollutants^3–5^.

Non-biodegradable colored organic pollutants (COPs) with genotoxic, mutagenic, and carcinogenic properties in wastewater are harmful to the environment and ecology. These pollutants hinder light penetration in water and impair photosynthesis in aquatic organisms^6,7^. Thus, developing an efficient, cost-effective technique to degrade COPs in wastewater is crucial.

Water treatment involves physicochemical and biological methods^8,9^. Advanced technologies like adsorption, advanced oxidation (by efficient materials such as peroxodisulfate (PDS), peroxomonosulfate (PMS) or hydrogen peroxide (H_2_O_2_)), flocculation, and filtration, along with innovative approaches like microbiological and electro-catalytic methods, have gained attention for water treatment^10–15^. Recent advances in environmental remediation have introduced novel techniques such as electrochemical and electro-phentonic methods for treating and quantifying COPs, offering efficient and precise approaches for their removal and measurement^16–18^. However, the complex chemical structure of some COPs makes their complete removal challenging using conventional methods like ion exchange, solvent extraction, chemical precipitation, and physical adsorption^19^. Although progress has been made in removing COPs from water, more efficient methods are needed. Nanotechnology have revolutionized pollutant removal techniques^20,21^. Catalytic degradation has emerged as a viable option for eliminating COPs from wastewater^22–25^.

The rapid growth of industry and population has led to energy shortage and environmental pollution. To ensure long-term and sustainable development, there is a need for environmentally friendly and renewable technologies^26^. Photocatalysts and nanostructured heterogeneous catalysts have shown promise in utilizing solar energy for green fuel production and pollutant degradation^27,28^. Efficient separation of photogenerated electron–hole pairs in semiconductor photocatalysts has been the subject of various proposed strategies. These include doping, metal loading, and introducing heterojunctions. Among these strategies, the engineering of heterojunctions in photocatalysts has emerged as a highly promising approach. It has been proven to be one of the most effective and feasible methods for preparing advanced photocatalysts, enabling the spatial separation of electron–hole pairs^29^. These photocatalysts come in various forms^30^. Some of those including Type I, Type II, Z-scheme and S-scheme have been introduced as heterojunction materials with more efficient in charge separation. Despite efforts to enhance their efficiency, their practical applications are limited due to low photocatalytic activity^27–30^.

Despite the high activity of homogeneous catalysts, the performance of any catalyst dissolved in water implies that ultimately the catalyst itself will become a secondary pollutant. Using solid, heterogeneous, recyclable, and waste-derived catalysts is crucial for preventing the generation of secondary pollutants^31^. These offer benefits like low toxicity, high efficiency, selectivity, short reaction times, and mild conditions for degrading dyes and pollutants. Proper utilization of such catalysts is crucial for a cleaner environment^22,32,33^. Metal–Organic Frameworks (MOFs) have become popular as catalyst support materials due to their high surface area, non-toxicity, low cost, customizable morphology, and impressive optical and chemical properties, as well as their recyclability and reusability^33–36^.

In recent years, MOFs have been extensively studied in environmental chemistry^37–39^. These MOFs, composed of organic linkers like terephthalic acid (TPA) and various metal active sites (gold, iron, zinc, copper, silver, etc.), have proven to be effective catalysts for the degradation of organic pollutants (COPs) in water^40–42^. Also, various metal–organic frameworks (MOFs) have been explored for catalyzing the oxidation of organic pollutants, activated by compounds like PDS, PMS, or H_2_O_2_. Bimetallic MOFs surpass single-metal ones, improving oxidation efficiency, catalytic activity, stability, and control in peroxide activation^14,15^.

Recent research has outlined the utilization of metallic nanoparticles, such as palladium^43,44^, copper^45,46^, nickel^47,48^, gold^49,50^, silver^51–53^, etc., and other elements, integrated into heterogeneous solid structures. These structures are specifically engineered for catalytic processes and the removal of color organic pollutants (COPs) like methylene blue, 4-nitrophenol, and methyl orange. Additionally, they are designed for recyclability and serve as efficient catalysts. Silver nanoparticles (Ag NPs) stand out due to easy synthesis, reactivity, and affordability. However, their tendency to aggregate limits catalytic efficiency^9^. Also, silver MOFs offer high biocompatibility, biodegradability, and antibacterial potential, making them valuable for antibacterial treatments. With lower cytotoxicity and superior stability compared to other metals, silver is utilized in drug delivery, medical imaging, diagnostics and water treatment. To date, researchers have documented numerous studies exploring the application of silver metal in both water treatment methods and the catalytic reduction of COPs^9,41,51–53^.

Among MOFs, MIL-101(Cr), a chromium-based MOF, boasts a high surface area, large pores, and robust stability^54^. Featuring unsaturated Lewis acid sites, it finds diverse applications in adsorption, gas storage, separation, and catalysis due to its unique physicochemical properties and structural attributes. It has been chosen as the framework material for developing composites to catalytic degradation of COPs. MOFs offer notable advantages but encounter challenges in catalytic processes due to their high band gaps. To address this limitation, the heterojunction method, involving the combination of narrow band gap semiconductors, has been employed to enhance electron transfer efficiency and decrease recombination rates, thereby improving catalytic performance. Silver, particularly silver nanoparticles and compounds such as Ag_3_PO4, Ag_2_O etc., are highly regarded for its potential in water treatment through catalytic (or photocatalytic) degradation processes. MIF-101 demonstrates promise for doping with silver nanoparticles, thereby enhancing MIL-101's effectiveness as a catalyst for degrading COPs. However, research on the application of Ag-MIL-101 remains limited, particularly concerning the removal of COPs. This study introduces a novel approach involving a silver-doped MIL-101 catalyst for removing COPs like MB, 4-NP and 4-NA under ambient conditions. Characterization techniques indicate that the incorporation of silver nanoparticles into MIL-101 results in reduced band gaps, facilitating electron transfers and enhancing catalytic efficiency in the degradation of COP compounds.

**Figure 1:**
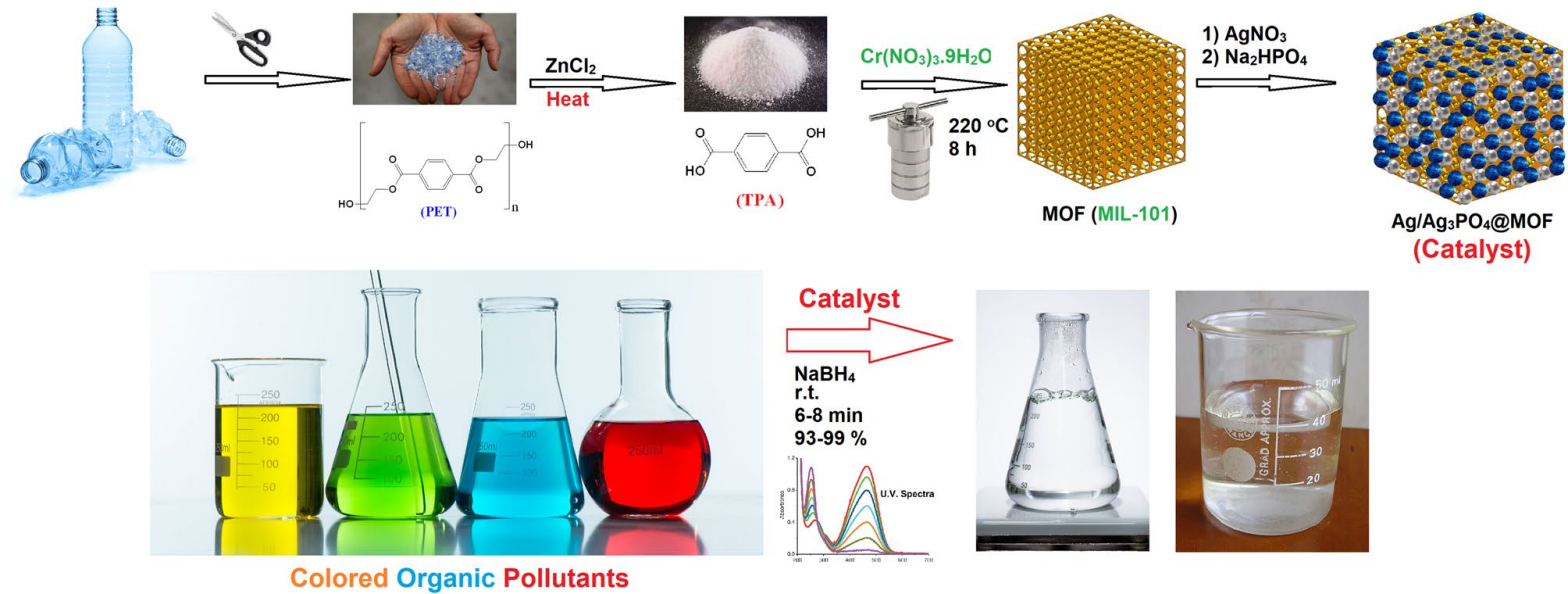
Synthesis of MOF composite derived from depolymerized PET as an efficient catalyst for the degradation of colored organic pollutants.

TPA, a commonly used organic linker for connecting metal ions in MOFs, has been extensively studied by researchers. One cost-effective and readily available method for obtaining this linker is through the degradation of PET plastic waste. As an environmentally method, MIL-101 was synthesized from discarded plastic bottles instead of commercial TPA to minimize secondary pollutants production^55,56^. Ag/Ag_3_PO_4_@MIL-101, a silver nanoparticle-incorporating MOF composite, has been studied as an affordable, reusable, and eco-friendly catalyst for targeted COP degradation. The findings confirm the exceptional catalytic performance of Ag-MIL-101, along with its favorable recyclability and reusability (Fig. 1).

## Material and methods

### Chemicals and materials

We obtained all the chemicals (Cr(NO_3_)_3_·9H_2_O, ZnCl_2_·H_2_O, Urea, Ethylene glycol (EG), Dimethyl formamide (DMF), Ethanol (EtOH), Methylene blue (MB), 4-Nitrophenol (4-NP), and 4-Nitroaniline (4-NA)) from Merck and Sigma-Aldrich Chemical companies. These chemicals were of analytical grade and used without any further purification. The used plastic waste bottles in this study were obtained from a public recycling center. TPA, a crucial component, was derived from the degradation of discarded plastic bottles.

### Catalytic depolymerization of PET

With slight modifications, the described methods were utilized to prepare the required terephthalic acid for MIL-101 synthesis^56,57^. Obtained PET water bottles from recycling facilities were washed, dried, and divided into 5 × 5 mm fragments (total weight: 5 g). The fragments were placed in a 50 mL glass flask with 20 g of ethylene glycol, equipped with a reflux condenser and a thermometer. A catalyst of 0.25 g ZnCl_2_: Urea (molar ratio 1:6) was added. Glycolysis of PET was conducted for 30 min at 170 ± 3 °C under atmospheric pressure, using a magnetic stirrer and an oil bath. After cooling to room temperature, 1000 mL of cold distilled water was added, the pH was adjusted to 3–4 using H_2_SO_4_ (2 M) to obtain a white slurry, and the resulting product (TPA) was filtered to separate it from the residual PET pellets. The residual PET was dried at 70 °C until constant weight was achieved. PET conversion was calculated as 87% using the formula.

$$Conversion\, of\, PET\, (\mathrm{\%})=(\frac{initial\, weight\, of\, PET - weight\, of\, residual\, PET}{initial\, weight\, of\, PET})\times 100$$

### Preparation of MIL-101

Obtained TPA from the degradation of plastic waste bottles (the previous step) was used and MIL-101 was synthesized using the hydrothermal method^58,59^. Initially, terephthalic acid (3.32 g), Cr(NO_3_)_3_·9H_2_O (8.0 g), and hydrofluoric acid (0.8 mL) were added to 100 mL of deionized water. The resulting mixture was stirred for 0.5 h using a magnetic stirrer. Subsequently, the mixture was transferred into a Teflon-lined autoclave and heated at 220 ± 5 °C for 8 h, followed by slow cooling to room temperature (5 °C/min). The produced green-solid was separated by centrifugation at 4000 rpm and washed with hot water until the supernatant became colorless. After vacuum drying at 80 °C, crude MIL-101 was obtained. To further purify the product and remove any remaining TPA and Cr(NO_3_)_3_·9H_2_O, the crude MIL-101 was refluxed in dimethyl formamide (DMF) solvent for 12 h. The product was then thoroughly washed with ethanol at 40 °C for 2 h. Finally, after drying in an oven at 80 °C for 10 h, pure MIL-101 powder was obtained.

### Preparation of Ag-MIL-101 composite

According to the literature, Ag-MIL-101 composite was synthesized using an in-situ precipitation method^58^. In order to achieve Ag-MIL-101 with maximum silver immobilization on its surface while minimizing losses during washing procedures, MIL-101 (10 g) were mixed with different concentrations of silver nitrate (2–25 mL, 0.3 M). The degree of silver loss was assessed through atomic absorption analysis, and eventually, the ideal conditions were employed for fabricating the optimized ratio of Ag on MIL-101. In optimized conditions, pre-synthesized MIL-101 (10 g) was added to deionized water (50 mL) and mixed at room temperature for 0.5 h. Subsequently, a solution of AgNO_3_ (10 mL, 0.3 M) was slowly added to the mixture while stirring. Dropwise addition of a solution of Na_2_HPO_4_·12H_2_O (10 mL, 0.1 M) was then carried out, followed by vigorous stirring in the dark condition for 12 h. The resulting product was thoroughly washed with deionized water (3 × 50 mL), ethanol (3 × 50 mL) and subsequently dried under vacuum at 80 °C overnight. The resulting Ag-MIL-101 composite, exhibiting green color, was designated as Ag-MIL-101.

### Characterization methods

In this study, the monomer TPA, derived from PET decomposition, 4-NP, and 4-aminophenol (4-AP) were characterized using ^1^H-NMR spectroscopy. The spectra were recorded in DMSO-*d*_*6*_ (or CDCl_3_) with a Bruker Avance DRX-400 MHz spectrometer, using tetramethylsilane (TMS = 0.00 ppm) as the internal standard. Also, FT-IR spectroscopy was employed to identify the linkages and functional groups present in the MOFs. The spectra were recorded using a JASCO 6300 spectrophotometer in the wavenumber range of 400–4000 cm^−1^, with KBr as the matrix. The crystal structure of MOFs were investigated using the D8-ADVANCE XRD instrument (Bruker, Germany) and Cu-Kα radiation. The scanning range was set from 10 to 90° at a rate of 2°/min. The surface morphology, average particle size and elemental composition of the synthesized samples were analyzed using the TESCAN BRNO field-emission scanning electron microscope (FE-SEM) at 15.0 kv, along with energy-dispersive X-ray spectroscopy (EDS). Transmission electron microscopy (TEM) images obtained from the Philips EM 208S instrument were used to further examine the sample morphology. Additionally, the progress of COP reduction reactions was monitored using a UV–Vis spectrophotometer equipped with a quartz cell, and absorption measurements were recorded to evaluate changes over time.

### Catalytic degradation of COPs

To assess the effectiveness of the synthesized Ag-MIL-101 catalyst, a catalytic degradation test was performed using MB, 4-NP and 4-NA as model COPs. In the experimental procedure, 1 × 10^–3^–15 × 10^–3^ g of Ag-MIL-101 were added to a 50 mL aqueous solution containing 50 ppm of COP (MB, 4-NP or 4-NA). In an effort to diminish the surface adsorption impact of COPs by the catalyst, the solution underwent agitation using a magnetic stirrer, allowing for a thorough examination of changes in adsorption intensity. The results indicated a marginal reduction in the magnitude of adsorption changes, with the intensity remaining unchanged following 20 min of agitation. Therefore, in each reaction, the reaction mixture was initially stirred for 30 min to ensure effective interaction between the catalyst and the COP, thereby establishing an adsorption–desorption equilibrium between them. Then, a 0.1 M NaBH_4_ solution (15–40 mL) was added to the reaction mixture. The reaction mixture was continuously stirred using a magnetic stirrer at room temperature throughout the duration. At regular intervals, 2.5 mL samples of the reaction solution were taken and rapidly diluted with 5 mL of water. The catalyst was subsequently separated from the mixture by centrifugation. The progress of color degradation was quantified using UV–Vis spectroscopy, specifically by measuring absorbance changes at the corresponding maximum wavelength (Table 1). The objective was to optimize the degradation conditions of the COPs catalyst by investigating factors such as reaction time, sodium borohydride dosage, catalyst efficiency, recovery, and reusability.

**Table 1:** The maximum wavelength of each COP used in catalytic degradation reactions in the presence of NaBH_4_ and Ag-MIL-101 catalyst.

COPs	MB	4-NP	4-NA
λ_max_ (nm)	663	400 (317)	380

## Results and discussion

### Molecular structure

**Figure 2:**
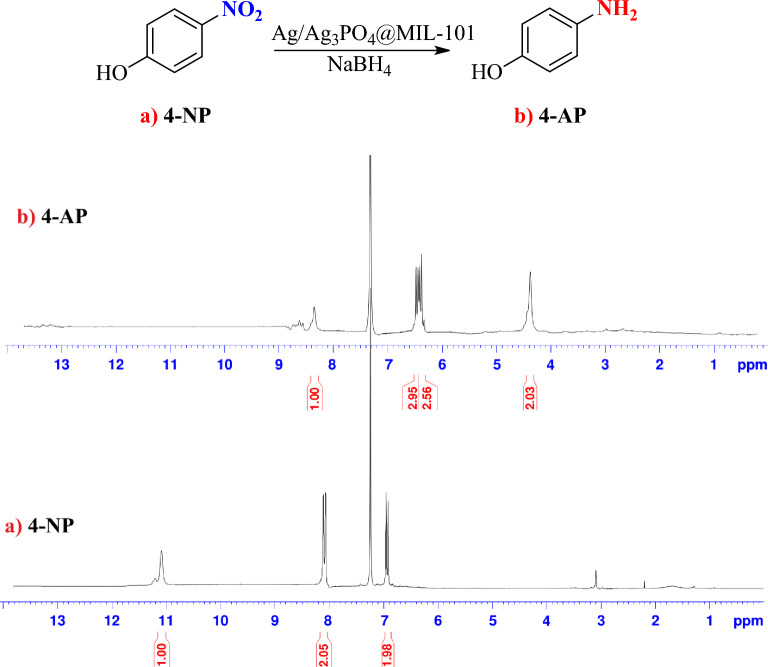
^1^H-NMR of 4-NP and produced 4-AP.

The TPA produced in this study was purified and its identity was confirmed using the ^1^H-NMR technique. The ^1^H-NMR spectrum (400 MHz, DMSO-*d*_*6*_) showed peaks at δ = 13.28 ppm (for –OH) and 8.05 ppm (for aromatic C-H). Also, the structural identity of 4-NP and 4-aminophenol (4-AP) (the reduced product of 4-NP) were determined and compared using ^1^H-NMR spectroscopy (Fig. 2). After the completion of the reduction reaction, the reaction solution was transferred to a separating funnel and extracted with diethyl ether. Subsequently, the resulting mixture underwent purification via packed column chromatography; 4-NP: ^1^H-NMR (400 MHz, CDCl_3_): 6.91 (d, 2H), 8.17 (d, 2H), 11.14 (s, 1H) (-OH) ppm; 4-AP: ^1^H-NMR (400 MHz, CDCl_3_): 4.37 (s, 2H) (-NH_2_), 4.22 (d, 2H), 4.25 (d, 2H), 8.38 (s, 1H) (-OH) ppm.

**Figure 3:**
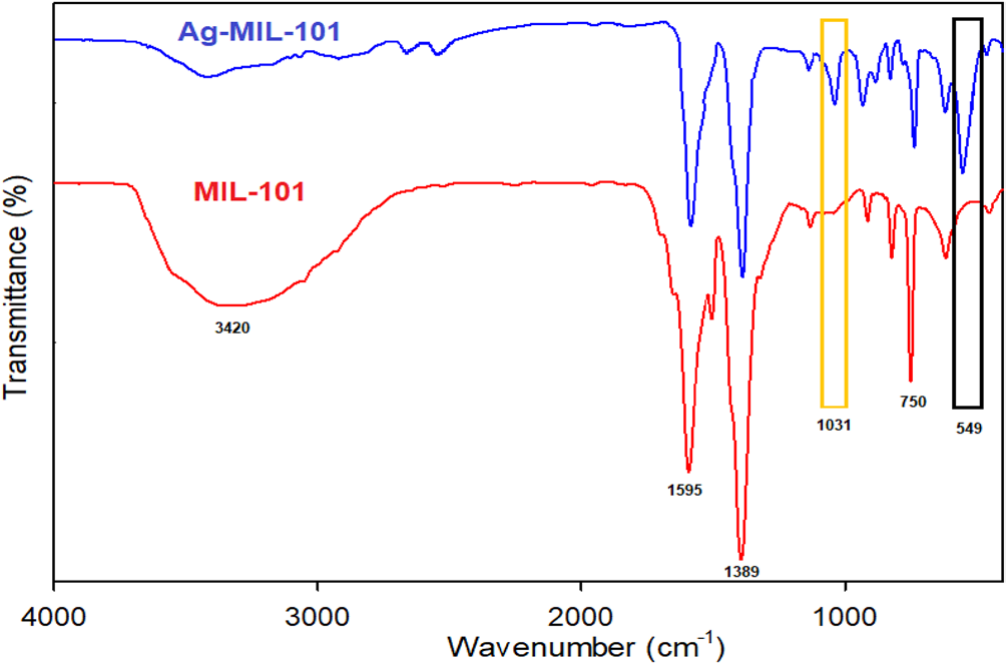
FT-IR of the synthesized MIL-101 and Ag-MIL-101.

The samples were analyzed using FT-IR spectroscopy. To facilitate a better and easier comparison, the results of the infrared analyses for the two compounds investigated in this research, MIL-101 and Ag-MIL-101, were placed side by side (Fig. 3). Considering the presence of similar functional groups in these two compounds, it is expected that the IR spectra of MIL-101 and Ag-MIL-101 would show significant similarities. The comparison of the two IR analyses in the image confirms this expectation. Furthermore, the results indicate that the incorporation of Ag_3_PO_4_ onto the surface of MIL-101 did not significantly alter the structure and bonds of MIL-101. A more detailed examination of the IR spectra of MIL-101 and Ag-MIL-101 reveals overlapping absorption bands at wavelengths of 3460, 1595, 1389, 1125, 920, 815, 750, 612, and 549 cm^−1^. The broad band near 3460 cm suggests the presence of crystalline water in the sample^59^. An intense band at around 1389 cm^−1^ indicates O-C-O symmetry. Additionally, the absorption band at 1595 cm^−1^ is related to the vibrational anisotropy of the dicarboxylate organic ligand^58,59^. The absorption at 750 and 920 cm^−1^ are associated with the C-H bands^60^. The characteristic band at 612 cm^−1^ corresponds to Cr–O stretching^58^. Importantly, compared to MIL-101, the absorption spectrum of the Ag-MIL-101 compound exhibits two additional and novel absorption bands at wavelengths of 1031 and 549 cm^−1^, which can be attributed to the presence of the phosphate functional group. The absorption bands near 1031 and 549 cm^−1^ correspond to P–O stretching and O–P–O bending vibrations of PO_4_^3−^, respectively^58,61^. The presence of these bands indicates the successful manufacturing process of the Ag-MIL-101 composite. Moreover, the production process of the Ag-MIL-101 composite did not cause any noticeable changes in the preexisting bonds and functional groups.

In conclusion, the samples were analyzed using FT-IR spectroscopy. The IR spectra of MIL-101 and Ag-MIL-101 show significant similarities, confirming the presence of similar functional groups. The addition of Ag_3_PO_4_ did not result in significant changes in the bonds of MIL-101. The IR spectra of MIL-101 and Ag-MIL-101 exhibit overlapping absorption bands, indicating the presence of specific functional groups. Additionally, the absorption spectrum of the Ag-MIL-101 composite shows two additional absorption bands related to the phosphate functional group, confirming the successful manufacturing process without causing noticeable changes to the preexisting bonds and functional groups.

### Crystalline and phase structure

**Figure 4:**
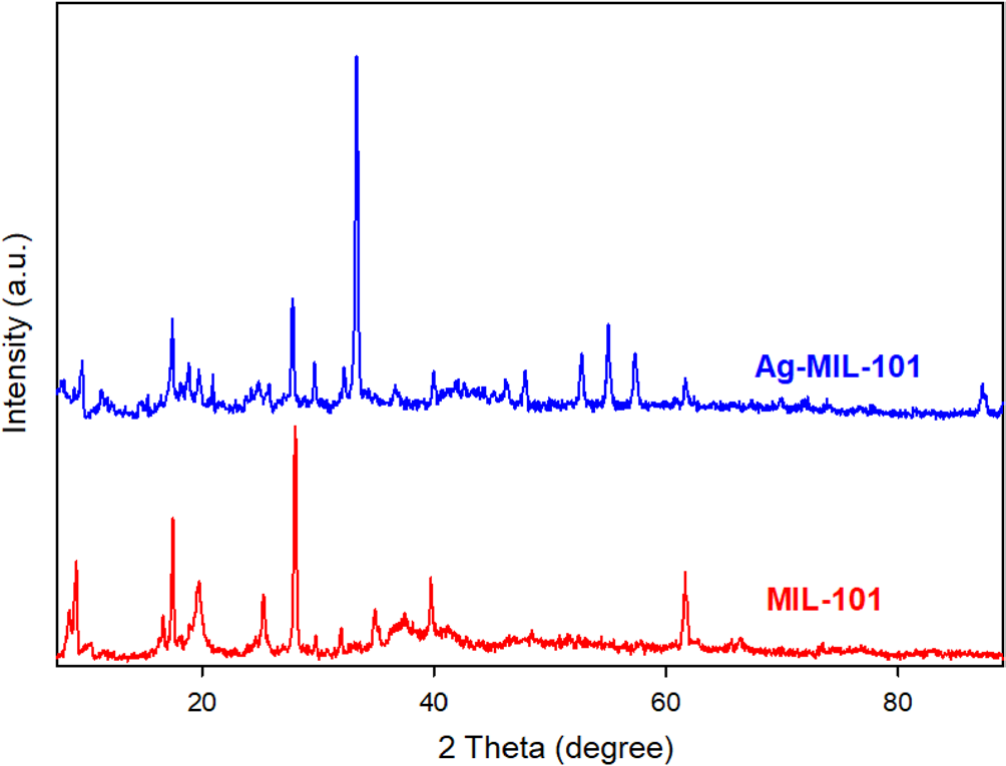
XRD of MOFs (MIL-101 and Ag-MIL-101).

XRD analysis has proven to be a valuable tool for confirming the formation and stability of NPs in MOFs. In this study, the representative XRD pattern of Ag-MIL-101 was compared to that of MIL-101, as shown in Fig. 4. Despite the adsorption of Ag_3_PO_4_ and subsequent reduction to Ag NPs, the crystallinity of MIL-101 remained intact. However, there was a noticeable shift in the peak positions following the adsorption of Ag_3_PO_4_. Fortunately, the XRD analysis confirmed that the as-synthesized Ag-MIL-101 possesses a structural arrangement similar to that of MIF-101 (Fig. 4). The crystal structures of MIL-101 and Ag-MIL-101 were found to be isostructural to each other and exhibited similar porosity. In these structures, the equatorial or axial sites of each Cr^3+^ ion are coordinated by O atoms from TPA unit linkers resulting in the formation of 3D MOFs. The addition of Ag_3_PO_4_ to MIL-101 leads to a noticeable darkening of its green color, while the crystalline structure remains unaffected, signifying the compatibility between these two materials. The slight displacement of diffraction peaks corresponding to MIL-101 can be attributed to the presence of Ag_3_PO_4_. To investigate the crystal structure and phase composition of the samples, X-ray diffraction (XRD) analysis was employed. The XRD pattern of the MIL-101 sample is depicted in Fig. 4. The obtained XRD peaks at 2θ angles of 5.2, 8.5, 9.1, 10.3, and 16.5° are in agreement with the literature, which confirms the presence of MIL-101 in the samples^58,62,63^. Moreover, the XRD pattern of the synthesized MIL-101 indicates its highly crystalline nature. The combined XRD patterns of MIL-101 and Ag-MIL-101 show distinctive peaks from MIL-101, Ag_3_PO_4_, and Ag nanoparticles, confirming successful synthesis of the composite. Comparative analysis reveals similar XRD patterns, except for the appearance of novel peaks at 2θ = 46.1 and 47.7, attributed to Ag nanoparticles, and peaks at 2θ = 52.6, 55.0, and 57.2, attributed to Ag_3_PO_4_. Importantly, no significant shifts in MIL-101 peaks are observed in the XRD patterns of composites with Ag, Ag_3_PO_4_, and MIL-101. This suggests that incorporating Ag_3_PO_4_ does not affect the crystal structure of MIL-101.

### Morphology and elemental composition

**Figure 5:**
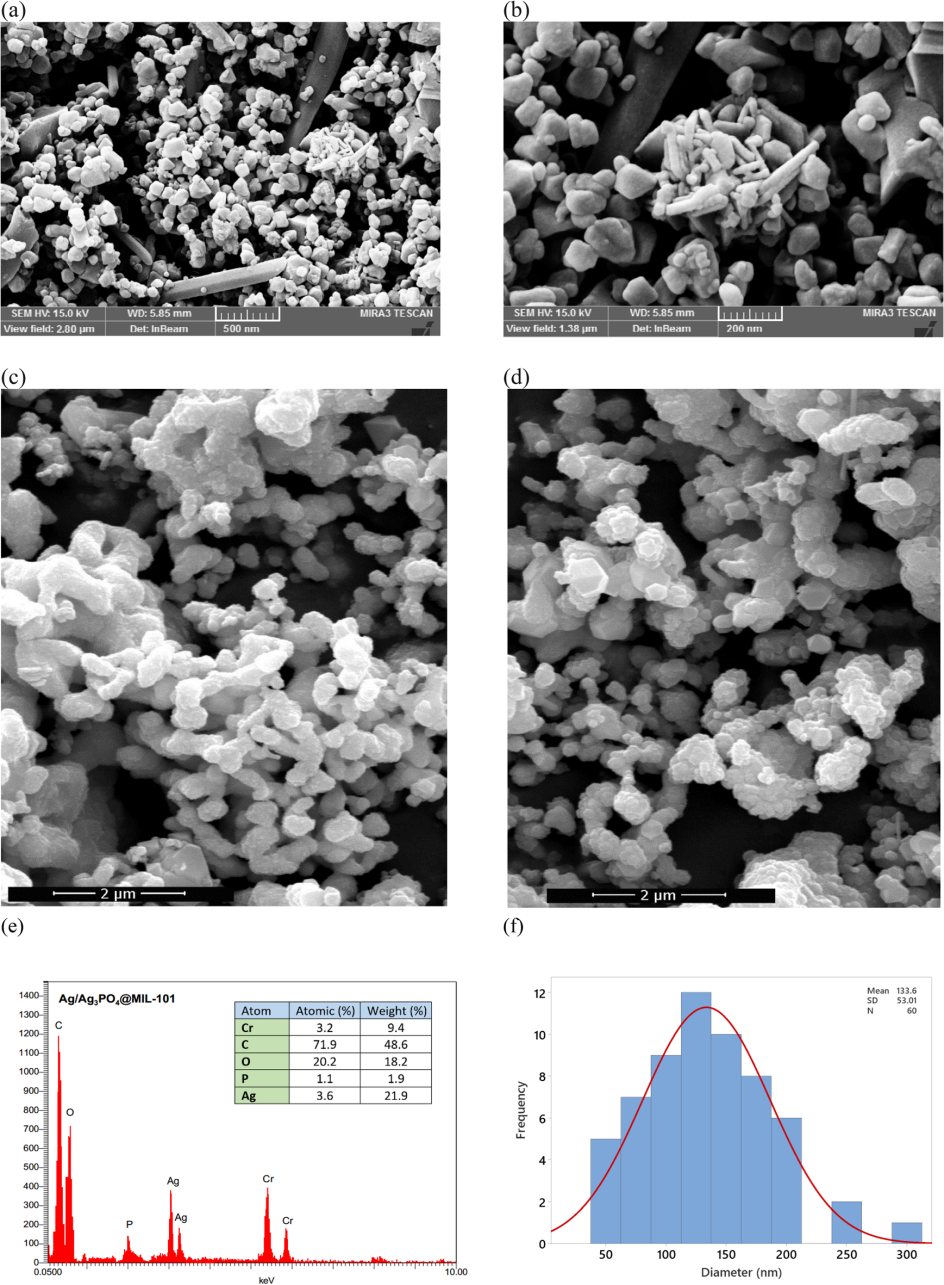
FE-SEM of prepared Ag-MIL-101 (**a**) and (**b**); FE-SEM of MB-treated recycled Ag-MIL-101 (**c**); FE-SEM of 4-NP-treated Ag-MIL-101; EDS analysis of Ag-MIL-101; and corresponding histogram of particle size distribution for synthesized Ag-MIL-101.

In this study, morphological and elemental analysis of the prepared Ag-MIL-101 compound samples was carried out using SEM, EDS, and TEM techniques. The SEM image (Fig. 5a,b) demonstrates the nano-particulate nature of Ag-MIL-101, showing distinct inclined aggregations and variations in particle sizes. Moreover, the image provides a detailed visualization of the geometric and octahedral structure of Ag-MIL-101. The SEM images depicted the Nano particulate nature of Ag-MIL-101 and its distinctive aggregations, while also highlighting the geometric and octahedral structure. Additionally, the particle size distribution analysis reveals a mean diameter of 133.6 ± 0.5 nm for the Ag-MIL-101, providing quantitative information about their size distribution. Furthermore, following the degradation and reduction of methylene blue and 4-nitrophenol, SEM images of the treated and recycled catalyst can be observed in Fig. 5c,d. The chemical composition of the Ag-MIL-101 compound was determined through energy dispersive spectroscopy (EDS) analysis. The presence of carbon (C), oxygen (O), phosphorus (P), silver (Ag), and chromium (Cr) was unequivocally validated and the composition of the catalyst is visually presented in Fig. 5e, accompanied by an EDS pattern that provides a comprehensive quantitative analysis of the constituent elements, displaying the percentages of elements as atomic and weight percentages. Also, corresponding histogram of particle size distribution for synthesized Ag-MIL-101 was shown in Fig. 5f.

**Figure 6:**
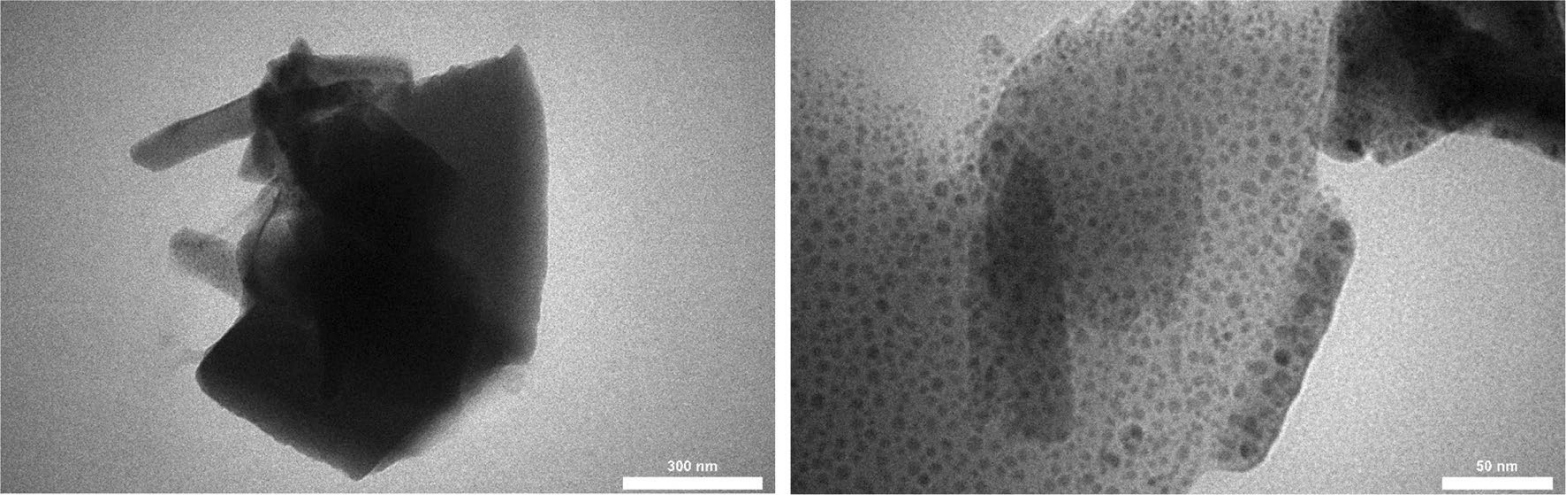
TEM of Ag-MIL-101.

The TEM characterization of Ag-MIL-101, as depicted in Fig. 6, offers a comprehensive understanding of the material's structural characteristics. The TEM image demonstrates the homogeneous distribution of metallic units within the MOFs, highlighting their uniformity. The presence of dark dots on the surface of MIL-101 indicates the presence of silver nanoparticles (Fig. 6). These silver nanoparticles facilitate efficient electron transfer between energy levels, thereby improving the speed and efficiency of the catalytic reaction.

### Optical properties

**Figure 7:**
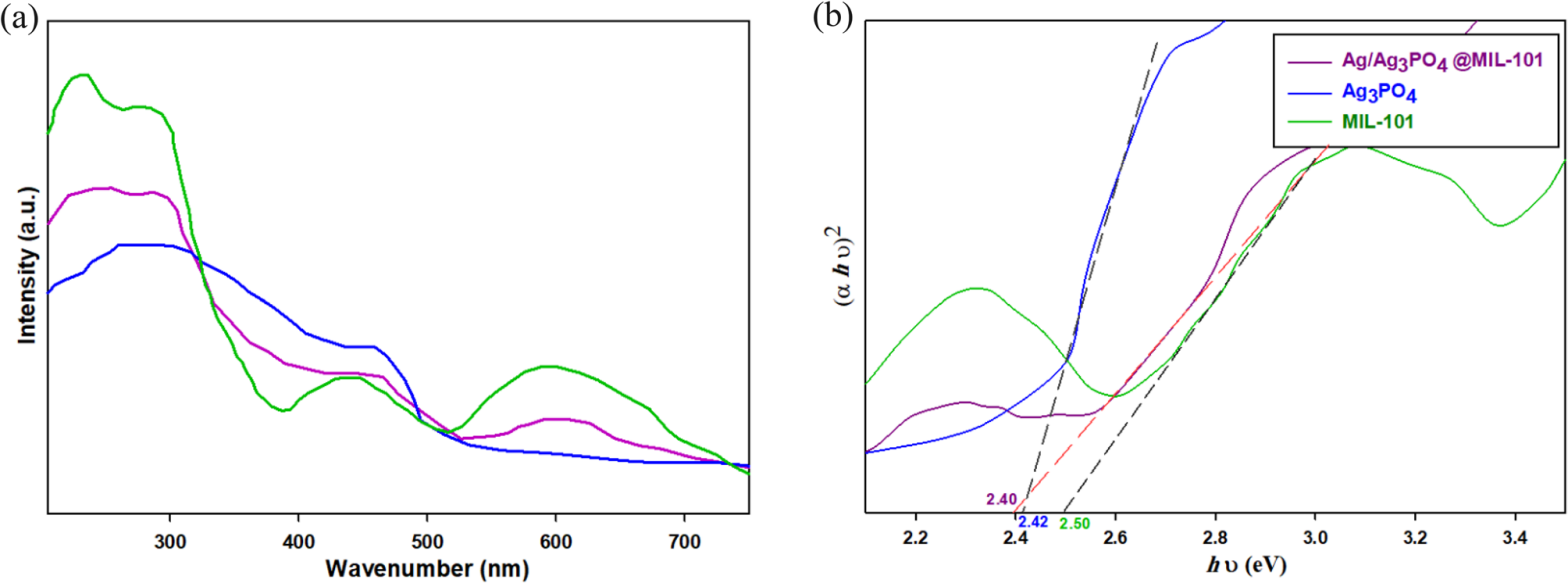
(**a**) UV − vis and (**b**) band gap energy spectra of MIL-101(Cr), Ag_3_PO_4_, and Ag-MIL-101 composite.

The optical properties of materials are essential for their photocatalytic performance. Using UV–vis absorption spectroscopy, we investigated these properties in our study. Comparing the Ag-MIL-101 composite to MIL-101(Cr) and Ag_3_PO_4_, we observed a shift in the absorption edges towards longer wavelengths. This shift indicates enhanced absorption in the visible range, improving photocatalytic performance. We used the Kubelka − Munk algorithm to obtain band gap spectra for MIL-101(Cr), Ag_3_PO_4_, and Ag-MIL-101 composite. The measured band gaps were 2.50 eV for MIL-101(Cr), 2.42 eV for Ag_3_PO_4_, and 2.40 eV for Ag-MIL-101 composite. The smaller band gap of the composite suggests more efficient utilization of visible light, highlighting its potential for photocatalytic applications (Fig. 7).

### Optimal conditions for the degradation of COPs

The use of catalysts in chemical processes is crucial for enhancing speed and reaction efficiency. Increasing the catalyst dosage does not always improve efficiency and can lead to economic wastage. Therefore, optimizing catalyst dosage is crucial for environmental protection and cost-effectiveness. Initially, we investigated the importance of key compounds in the degradation of COP to determine the optimal reaction conditions (Table 2). Each type of COP (MB, 4-NP and 4-NA) was individually evaluated to establish the most appropriate conditions. The degradation of an equal amount of COP (50 mL, 50 ppm) was measured and assessed in all reactions. Initially, the degradation of the COP was examined in the presence of MOF and NaBH_4_ separately or simultaneously.

**Table 2:** The key role and simultaneous presence of Ag-MIL-101 and NaBH_4_ in the catalytic degradation of COPs.

Entry	Catalyst (g × 10^–3^)	NaBH_4_ (mL, 0.1 M)	MB Deg. (%)^a^	4-NP Deg. (%)	4-NA Deg. (%)
1	MIL-101 (5.0)	–	–	–	–
2	MIL-101 (5.0)	40	11	7	5
3	Ag-MIL-101 (5.0)	–	–	–	–
4	Ag-MIL-101 (5.0)	40	98	99	94
5	–	40	6	–	–
6	Ag_3_PO_4_ (5.0)	40	99	98	97
7	Ag_3_PO_4_ (5.0)	–	15	18	12
8^b^	Ag-MIL-101 (5.0)	–	37	32	26

Conditions: All reaction efficiencies were determined by measuring the degradation of COP solution (50 mL, 50 ppm) after 2 h of reaction time.

^a^Deg.: Degradation.

^b^The degradation of COPs was conducted under sunlight radiation, and the results were measured after 2 h reaction time.

The photocatalytic degradation reaction of the desired COPs was investigated in the presence of the Ag-MIL-101 composite, and the results are reported in Table 2 (entry 8). The photocatalytic capability of Ag-MIL-101 composite in degrading COP compounds was investigated under light radiation at room temperature. A solution containing COP compound (50 mL, 50 ppm) and Ag-MIL-101 composite (5 × 10^–3^ g) was exposed to sunlight, and the change in UV–vis absorption of COPs was measured. Results demonstrated that, while the photocatalytic reaction had its advantages, the efficiency of the composite in the presence of NaBH_4_ far exceeded the degradation of COPs under light radiation (Table 2, entry 8). To examine the impact of light absorption on the photodegradation of the desired COPs, photolysis of these compounds was investigated using sunlight without catalyst. However, after 120 min, no significant alteration in the absorption intensity at the maximum wavelength of the COPs was observed.

The results demonstrated a significant decrease in COP degradation in the absence of either NaBH_4_ or Ag-MIL-101, particularly with prolonged reaction time. These findings highlight the necessity of both compounds (NaBH_4_ and Ag-MIL-101) for achieving satisfactory efficiency in the degradation of COPs. Furthermore, it can be concluded that silver metal plays a crucial catalytic role in the COP degradation reaction. In the absence of catalyst, the COP degradation reaction fails to occur, potentially due to a significant discrepancy between the energy level of the reducing agent (NaBH_4_) and the acceptor (COP compound). The energy levels of these species remain distant, and the absorption intensity at λ_max_ remains relatively constant, suggesting limited color reduction and degradation. However, the inclusion of the Ag-MIL-101 composite as a catalyst in the reaction mixture creates an intermediate electronic level through the presence of silver metal. This enables the facilitation of electron transfer between energy levels by reducing the necessary distance for electron transfers. As evident from the obtained results in Table 2 (Entries 6 and 7), the Ag_3_PO_4_ compound can serve as an efficient catalyst for the degradation of COPs. Comparison of Entries 6 and 7 reveals that while Ag_3_PO_4_ has limited capability in degrading COPs, the addition of NaBH_4_ greatly enhances reaction efficiency and degradation rate. Sodium borohydride is thought to induce the reduction of silver ions and formation of Ag nanoparticles, enabling them to effectively catalyze the degradation of the desired COP compounds^9,64^. Considering our primary objective of conducting environmentally friendly processes and degrading environmental pollutants, we have overlooked application of Ag-MIL-101 composite as catalyst for the COP degradation reaction in the presence of NaBH_4_. Subsequently, all remaining reactions to find optimized conditions were conducted with the simultaneous presence of NaBH_4_ and Ag-MIL-101 (Table 3).

**Table 3:** Optimization of catalytic degradation conditions of COPs in the simultaneous presence of Ag-MIL-101 and NaBH_4_.

Entry^a^	Ag-MIL-101 (× 10^–3^ g)	NaBH_4_ (mL, 0.1 M)	MB	4-NP	4-NA
			Time (min)/Deg	Time (min)/Deg	Time (min)/Deg
1	15	30	5/98	4/97	8/95
2	12	30	3/99	3/96	8/94
3	10	30	3.5/98	3/98	7/95
4	7	40	3/97	3/97	7/95
5	5	40	3/96	4/98	7/93
6	5	30	3/97	4/97	8/93
7	5	20	3/98	4/97	15/88
8	5	10	3/97	5/97	15/82
9	4	10	4/96	**6/98**	–
10	3	10	**7/99**	7/90	–
11	2	10	8/90	15/86	–
12	1	10	15/88	20/83	–
13	4	30	–	–	**8/93**
14	3	30	–	–	15/87
15	2	30	–	–	20/80
16	1	30	–	–	30/72

^a^Optimum conditions are bolded.

The degradation of desired COPs (50 mL, 50 ppm) was conducted and examined in the presence of various doses of Ag-MIL-101 (1 × 10^–3^–15 × 10^–3^ g) and NaBH_4_ (0.1 M, 10–40 mL). The experimental results demonstrate the positive impact of increasing the dosage of Ag-MIL-101 and NaBH_4_ on the reaction rate and efficiency, confirming our initial expectations. Increasing the dosage of the Ag-MIL-101 and NaBH_4_ leads to an increase in the number of reaction sites on the surface, resulting in improved overall efficiency of COP degradation and apparent reaction rate. The results, indicate that the degradation efficiency of the investigated COPs is not significantly affected by the use of doses higher than 5 × 10^–3^ g of catalyst in each reaction. However, it should be noted that once the catalyst (or NaBH_4_) dosage surpasses a certain threshold, the catalytic degradation efficiency of COPs reaches a saturation point. The obtained results, which can be found in Table 3, provide valuable insights.

The obtained results demonstrate a clear correlation between the degradation of the target COP and the decrease in intensity of its maximum absorption wavelength. Notably, the maximum absorption wavelengths for MB, 4-NP, and 4-NA COPs are measured at 663, 400, and 380 nm, respectively. It is noteworthy that the 4-NP exhibited a maximum absorption at 317 nm. However, when combined with NaBH_4_, the maximum absorption peak of 4-NP shifted to 400 nm. This shift in absorption wavelength is attributed to the inherent bathochromic properties of the 4-NP compound^65^. Consequently, in the evaluation and assessment of the catalytic degradation reaction of 4-NP, the alterations in absorption intensity at 400 nm wavelength were utilized as the primary parameter. Table 3 presents a summarized overview of the specific values of Ag-MIL-101 and NaBH_4_ that were utilized to determine the optimal reaction conditions for the degradation of MB, 4-NP, and 4-NA COPs.

The results indicate that the degradation of the investigated COPs can be efficiently achieved within a short time (6–8 min) under optimized conditions, with an exceptional efficiency exceeding 93%. Also, it can be concluded that the Ag-MIL-101 composite can serve as a highly suitable catalyst for the efficient degradation of COPs present in real wastewater samples.

Subsequently, the reaction kinetics of each COP were evaluated under the identified optimal degradation conditions. In this study, we have concentrated on examining the kinetics of COP degradation reactions by specifically focusing on the reduction of absorption intensity at the corresponding maximum wavelengths. This choice was made based on the direct relationship between the change in absorption intensity and the percentage of degradation reaction progress. The following equation has been utilized in this study to determine the reaction efficiency of COP degradation in the presence of Ag-MIL-101:

$$Degradation (\%)=\left(\frac{[Ao] - [At]}{[Ao]}\right)\times 100$$

To analyze the degradation reaction kinetics of COPs, a concentration–time graph was prepared for the COPs. Due to the significantly higher concentration of NaBH_4_ (0.1 M) compared to COPs, the reduction process was investigated as a pseudo-first-order reaction. [C_o_] and [A_o_] represented the initial concentration and absorbance intensity, respectively. Similarly, [C_t_] and [A_t_] represented the concentration and absorbance intensity during the reaction. Using the equation and the absorbance-concentration relationship, a ln ([C_o_]/[C_t_]) vs. time graph was plotted. The data were fitted to a linear trend line to obtain the reaction rate constant (*k*_*app*_, min^−1^).

$$ln ({A}_{t}/{A}_{o})=ln ([{C}_{t}]/[{C}_{o}])= -kt$$

so:

$$ln ([{C}_{o}]/[{C}_{t}]) = +kt$$

**Figure 8:**
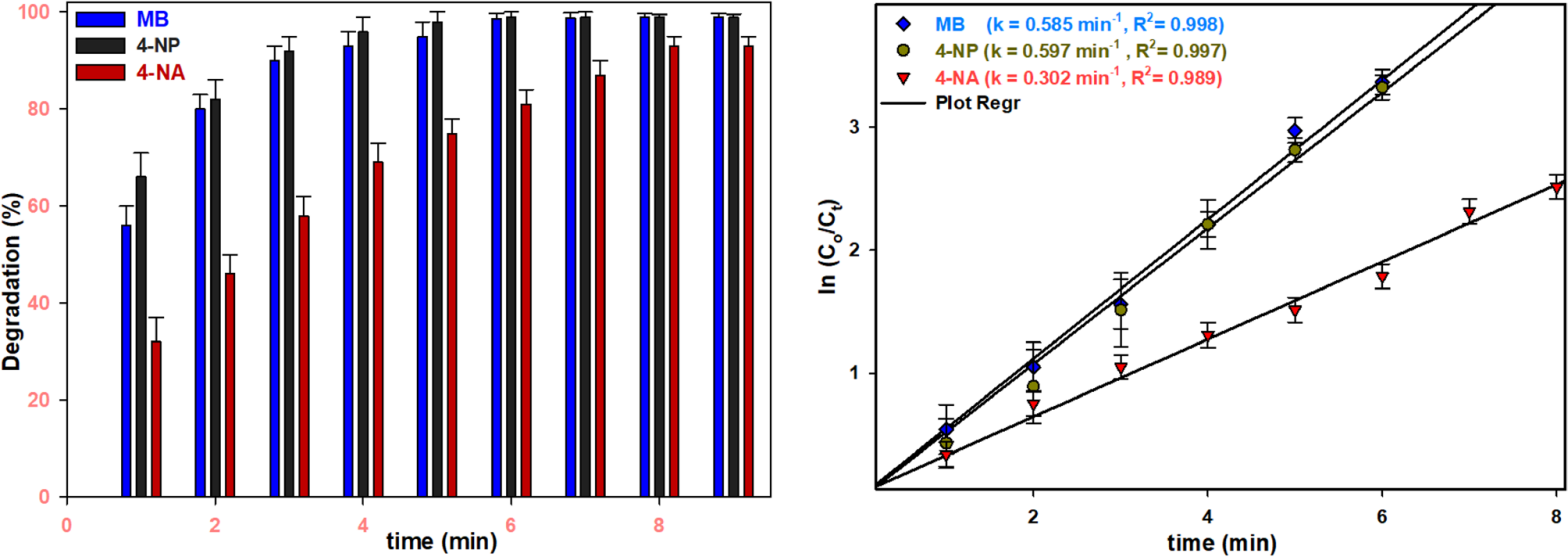
Kinetic study of degradation reaction of COPs (MB, 4-NP and 4-NA).

Under optimized conditions, the degradation reactions of COPs were examined with a specific emphasis on their kinetic behavior. The study revealed distinct rate constants for the degradation of various COPs: MB, 4-NP, and 4-NA exhibited rate constants of 0.58, 0.60, and 0.32 min^−1^, respectively (Fig. 8).

### Selective degradation of OPDs

The study aimed to examine the degradation behavior of COPs under specific experimental conditions. Equal quantities of two distinct COPs were introduced into a single batch and subjected to degradation under similar conditions at room temperature. The outcomes are detailed in Table 4. The findings indicate that the degradation process of MB and 4-NP lacks significant selectivity. However, when each of these pollutants is investigated alongside 4-NA, a substantial enhancement in the reaction's selectivity is observed.

**Table 4:** The catalytic and selective degradation of COPs in the presence of Ag-MIL-101.

Entry	COP-1 (25 mL, 100 ppm)	COP-2 (25 mL, 100 ppm)	COP-1 (Degradation %)	COP-1 (Degradation %)
1	MB	4-NP	95	91
2	MB	4-NA	98	72
3	4-NP	4-NA	92	76

Conditions: All reaction efficiencies were determined by measuring the degradation of COP solution (50 mL, 50 ppm), NaBH_4_ (20 mL, 0.1 M) and Ag-MIL-101 (4 × 10^–3^ g) after 20 min of reaction time.

### Proposed mechanism of COP degradation

**Figure 9:**
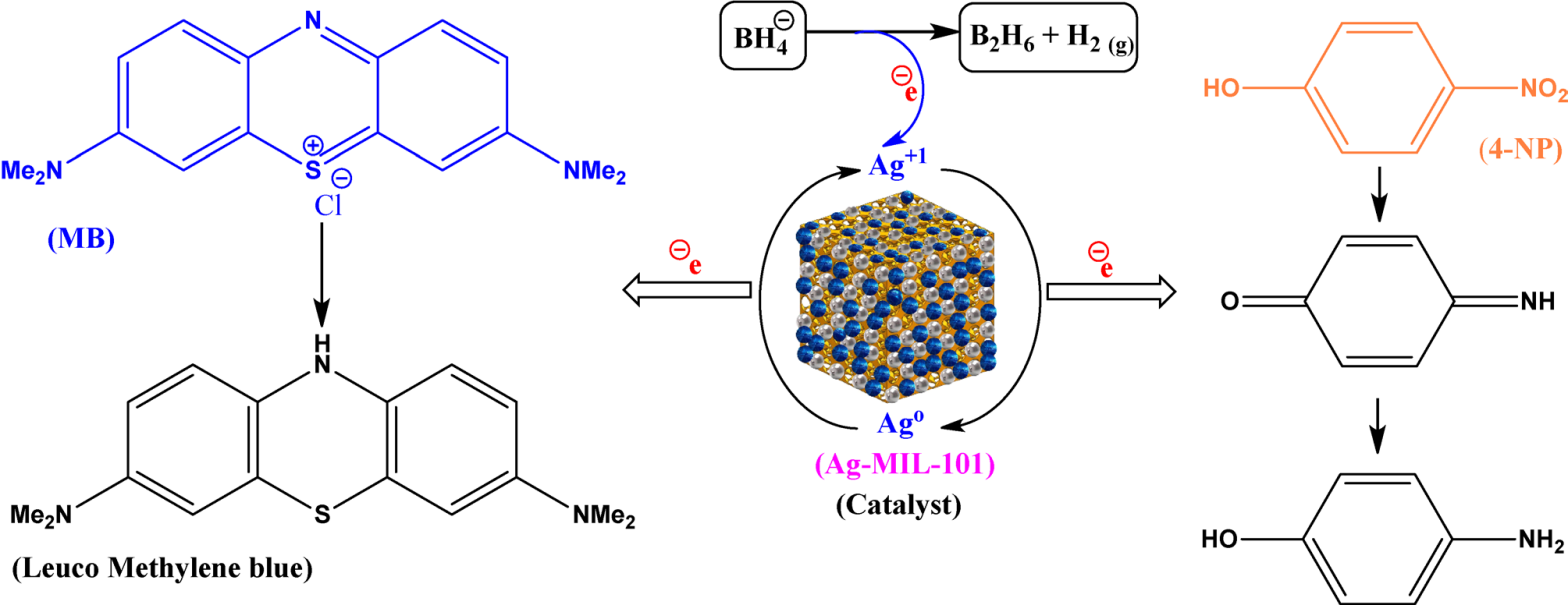
The proposed mechanism for the reduction of MB and 4-NP in the presence of Ag-MIL-101.

While a comprehensive investigation into the degradation mechanism remains outstanding, existing literature posits a potential mechanism for the reduction of COPs (MB and 4-NP), illustrated in Fig. 9^39,66,67^. Studies suggest that the incorporation of doped silver metal sites onto the MIL-101 framework initiates a reduction in energy band gaps, thereby narrowing the energy level gap between the lowest unoccupied molecular orbital (LUMO) and highest occupied molecular orbital (HOMO) levels. Consequently, this facilitates active interactions between the metal active sites and the dye pollutants, leading to a notable acceleration in the reaction rate.

In this study, our objective was to utilize Ag-MIL-101 as a novel and efficient catalyst in catalytic degradation of COPs in the presence of NaBH_4_ solution. The acquired outcomes were subsequently juxtaposed against the findings reported in comparable investigations, thereby elucidating the outstanding efficacy of the catalyst. It exhibited a notably elevated level of degradation progress, an accelerated reaction rate, and a reduced catalyst dosage-to-COP ratio. These findings underscore the superior benefits provided by this catalyst in comparison to alternative catalysts employed in prior studies (Table 5).

**Table 5:** Comparison of catalytic reduction of MB and 4-NP in the presence of various catalysts.

Catalyst (× 10^–3^ g)	COP (mL, ppm)	NaBH_4_ (mL, M)	Time (min)	*k*^c^ (min^−1^)	Refs.
MPD-Cu (0.4)^a^	MB (0.1, 200)	(0.66, 3)	5	1.44	^68^
Cu(NPs)/β-CCP (10)^b^	MB (2, 166)	(0.50, 0.04)	4	0.57	^69^
AgMoOS (10)	MB (100, 20)	(2.0, 0.2)	6	0.541	^70^
NC-AgNPs (50)	MB (2, 20)	(0.95, 0.005)	150	0.16	^71^
Ag-MIL-101 (3)	MB (50, 50)	(10, 0.1)	7	0.58	This work
Co/PCNS (0.1)	4-NP (2, 20)	(11, 0.125)	7	0.31	^72^
AgMoOS (10)	4-NP (100, 20)	(2, 0.2)	18	0.136	^70^
Cu-NP/C (4.0)	4-NP (1.5, 27.8)	(1.5, 0.02)	6	0.3	^73^
Cu/MC (0.5)	4-NP (6, 42)	(2, 0.5)	5	0.96	^74^
Ag-MIL-101 (4)	4-NP (50, 50)	(10, 0.1)	6	0.6	This work

^a^Magnetic polydopamine-Cu nanoflowers, ^b^Cu(NPs)/β-Chitin/dicalcium phosphate, ^c^Rate constant.

Furthermore, a comparison was made between the photocatalytic degradation results of methylene blue using various photocatalysts (Table 6).

**Table 6:** Comparison of photocatalytic reduction of MB in the presence of various catalysts.

Entry	Photocatalyst	Time (min)	Degradation (%)	Refs.
1	Cu/Ag/TiO_2_	120	20	^75^
2	CQD/TiO_2_/Fe_2_O_3_	180	86.5	^76^
3	CDS@C-mTiO_2_	35	95	^77^
4	Ag-MIL-101	120	37	This work

### Evaluating the Ag-MIL-101 catalyst's recyclability and reusability

**Figure 10:**
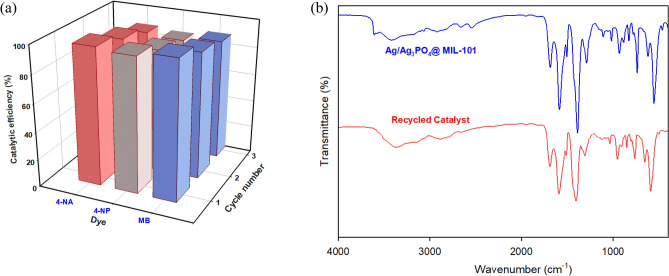
(**a**) Catalytic efficiency of recycled catalyst, (**b**) FT-IR spectra of catalyst before and after three catalytic cycles.

From an environmental perspective, catalysts that have better chemical stability, recyclability, and reusability are particularly important and also hold economic significance. These properties are crucial for the practical utilization of catalysts. Although the main objective of researchers in this study is the removal of pollutants and the presentation of efficient conditions for water treatment, it should be noted that the importance of catalyst recyclability in environmental preservation, waste reduction, and energy consumption cannot be underestimated. Therefore, in this study, we investigated the recoverability and reusability of the used catalyst. After the completion of the COP degradation reaction, the used catalysts were collected using filtration, centrifugation, washed, and dried overnight. The catalyst's efficiency in this study is directly impacted by the concentration of silver particles in its structure. Our findings indicate that the recycled catalyst significantly decreases in efficiency by approximately 10–15% after three recovery steps (Fig. 10a). These results emphasize the strong interactions between the silver component and the MOF matrix, which effectively prevent silver leaching during the recycling and cleaning processes. Consequently, this improves the stability and longevity of the catalyst, emphasizing its substantial implications in the respective domain. In addition, FT-IR analysis was performed on the catalyst before and after three cycles of experiments. Surprisingly, minimal differences were observed in the FT-IR pattern of the Ag-MIL-101 composite compared to the unreacted and fresh composite, suggesting that the functional groups remained unchanged throughout the three cycles (Fig. 10b).

## Conclusion

In this study, TPA was synthesized from PET bottles through solvothermal catalytic degradation, aiming to reduce plastic waste. The Ag-MIL-101 composite, synthesized using chromium and silver salts, was selected as the optimal catalyst for COP degradation based on factors such as degradation efficiency, reaction conditions, and catalyst recoverability. The catalytic ability of Ag-MIL-101 in the degradation of COPs (MB, 4-NP, and 4-NA) in the presence of NaBH_4_ was investigated. The results showed that under optimal conditions, the degradation of the examined COPs was achieved within 5–10 min with an efficiency of over 93%. The kinetics of the degradation reaction were evaluated, and the reaction rate constants for each COP was calculated. The Ag-MIL-101 catalyst demonstrated not only efficient COP degradation but also good recyclability and reusability, indicating its stability and excellent performance. Based on the advantages of the Ag-MIL-101 composite, its use in other catalytic reactions is recommended. This study presents an innovative approach to reducing environmental pollutants by utilizing plastic waste as a raw material for the production of a stable and recyclable catalyst. Further research is needed to optimize the process and evaluate its feasibility in large-scale applications. The study emphasizes the importance of using waste materials for sustainable solutions and provides an effective method to address environmental challenges. Our future research will focus on investigating photocatalytic degradation using MIL-101 composites for the degradation of COPs and pharmaceutical pollutants. This research not only highlights the significant role of MOF composites in water treatment but also opens new perspectives for the development of highly efficient catalytic systems based on MOFs. The preparation conditions of the Ag-MIL-101 composite could be evaluated in future research to enhance its photocatalytic efficiency for the degradation of COPs. By doing so, the photocatalytic properties and effectiveness of the composite in the photodegradation reaction can be improved.

## Data Availability

The datasets used and/or analyzed during the current study are available from the corresponding author at reasonable request.
